# Crestal bone loss and soft tissue changes in epicrestal versus subcrestal implants: An *in vivo* study

**DOI:** 10.6026/973206300223281

**Published:** 2026-06-30

**Authors:** Amit Siwach, Sanatomba Thingujam, Anil Sharma, Shrimant Raman, Reenu Sirohi, Shivani Saran

**Affiliations:** 1 Department of Prosthodontics and Crown & Bridge, Kalka Dental College, Meerut, Uttar Pradesh, India; 2 Department of Public Health Dentistry, Kalka Dental College, Meerut, Uttar Pradesh, India

**Keywords:** Crestal bone loss (CBL), dental implants, epicrestal placement, peri-implant tissues, subcrestal placement

## Abstract

The long-term success of dental implants depends on preservation of crestal bone and peri-implant soft tissue, yet the optimal depth of implant placement remains controversial. Therefore, it is of interest to compare clinical and radiographic outcomes of epicrestal and subcrestal implant placement at baseline, 6 months and 12 months. Groups showed comparable baseline conditions with no significant intergroup differences (p > 0.05), while subcrestal implants showed consistently lower marginal bone loss and probing depths over time. Although intragroup comparisons revealed significant bone loss progression (p < 0.001), all outcomes remained within acceptable clinical success criteria. Thus, we report that subcrestal implant placement offer a biological advantage in peri-implant tissue preservation despite statistically comparable outcomes.

## Background:

Dental implants have become one of the most predictable and widely accepted treatment modalities for the replacement of missing teeth, offering superior functional and aesthetic outcomes compared to conventional prostheses. Tooth loss not only compromises mastication and phonetics but also leads to progressive alveolar bone resorption and soft tissue collapse, ultimately affecting the patient's quality of life [1]. Although removable and fixed prostheses remain viable options, their limitations in retention, stability and long-term patient satisfaction have shifted the focus toward implant-supported rehabilitation as a more reliable alternative [2]. Over the past few decades, advancements in implant design, surface modifications and surgical protocols have significantly improved implant survival rates. However, long-term success is not solely dependent on osseointegration but also on the maintenance of peri-implant hard and soft tissues [3]. Crestal bone loss (CBL) and peri-implant soft tissue health are considered critical parameters for evaluating implant success. It has been well documented that the majority of crestal bone remodeling occurs within the first year after implant loading, with an average bone loss of up to 1.5 mm, followed by an annual bone loss of approximately 0.2 mm [4]. One of the key factors influencing peri-implant tissue stability is the vertical positioning of the implant relative to the crestal bone. Implants can be placed at the level of the alveolar crest (epicrestal placement) or below the crest (subcrestal placement) [5]. Epicrestal placement offers advantages such as easier surgical access, favorable load distribution and reduced technical complexity [6]. However, the proximity of the implant-abutment junction to the crestal bone may increase the risk of early bone remodeling and peri-implant inflammation due to microbial colonization at the microgap [7]. In contrast, subcrestal implant placement has been proposed as a strategy to preserve marginal bone levels and improve soft tissue stability. By positioning the implant deeper within the bone, this approach may enhance the establishment of biologic width, reduce crestal bone resorption and provide better aesthetic outcomes, particularly in areas with thin gingival biotype [8]. Additionally, subcrestal placement may decrease the likelihood of implant exposure and peri-implantitis by creating a more favorable environment for soft tissue adaptation. The concept of biologic width plays a crucial role in peri-implant tissue dynamics. Around implants, the biologic width is typically greater than that around natural teeth, ranging between 3-4 mm and consists of junctional epithelium and connective tissue attachment. Any disruption in this dimension can lead to bone remodeling as the body attempts to re-establish a stable soft tissue seal. Therefore, implant placement depth, prosthetic design and soft tissue characteristics collectively influence peri-implant health and long-term outcomes [9]. Radiographic evaluation, particularly using intraoral periapical radiographs and cone beam computed tomography (CBCT), plays an essential role in assessing crestal bone levels and monitoring peri-implant changes over time. CBCT provides three-dimensional visualization, allowing accurate assessment of bone quantity, quality and anatomical structures, thereby improving treatment planning and post-operative evaluation [10]. Clinical parameters such as probing pocket depth, bleeding on probing and keratinized mucosa width further complement radiographic findings in evaluating implant success. Despite numerous studies investigating implant placement depths, the literature remains inconclusive regarding the superiority of epicrestal versus subcrestal positioning, particularly in relation to combined hard and soft tissue outcomes. Variations in study design implant systems and patient-related factors contribute to this lack of consensus, highlighting the need for controlled clinical studies [11]. Therefore, it is of interest to evaluate the clinical and radiographic differences in CBL and soft tissue changes associated with epicrestal and subcrestal implant placement.

## Methodology:

The present *in vivo* randomized comparative clinical study was conducted to evaluate CBL and soft tissue changes in implants placed at epicrestal and subcrestal levels. Patients requiring implant placement were first informed about all treatment options, including the advantages and limitations of implant therapy and only those who were willing to undergo surgery and attend follow-up visits were included. Selection of patients was based on predefined inclusion and exclusion criteria. Healthy individuals aged between 20-50 years with sufficient alveolar bone and no local infection were included, while patients with systemic diseases, poor oral hygiene, parafunctional habits, or habits like smoking and alcohol abuse were excluded. A total sample size of 20 implants was determined based on statistical calculations, with 10 implants in each group. Patients were randomly divided into two groups using a lottery method: the epicrestal group, where implants were placed at the level of the crestal bone and the subcrestal group, where implants were placed 1.5-2 mm below the crestal bone level. Before surgery, all patients underwent a detailed diagnostic evaluation including medical and dental history, clinical examination and laboratory investigations such as blood tests to ensure fitness for surgery. Oral health was optimized by treating any existing infection, caries, or periodontal disease. Radiographic assessment using CBCT was performed to evaluate bone quality, quantity and anatomical landmarks and to determine the ideal implant size and position. A diagnostic wax-up and surgical stent were prepared to guide accurate implant placement. The surgical procedure was carried out under strict aseptic conditions. Local anesthesia using 2% lignocaine with adrenaline was administered. A crestal incision was made, followed by elevation of a full-thickness mucoperiosteal flap to expose the alveolar bone. Osteotomy preparation was performed using sequential drilling under saline irrigation according to the manufacturer's protocol. In the epicrestal group, the implant was placed at the crestal bone level, whereas in the subcrestal group, it was placed 1.5-2 mm below the crest. Primary stability was checked and a healing abutment or cover screw was placed. The flap was repositioned and sutured and patients were given post-operative instructions along with antibiotics, analgesics and mouth rinse. After a healing period of approximately 3 months, a second-stage surgery was performed and healing abutments were placed to allow soft tissue conditioning. Final prosthetic crowns were then fabricated and cemented. Baseline data were recorded at this stage and patients were recalled for follow-up at 6 months and 12 months after implant placement. Clinical evaluation included assessment of peri-implant soft tissue parameters such as probing pocket depth, which was measured at different surfaces of the implant using a William probe. Radiographic evaluation was performed using CBCT at baseline, 6 months and 12 months to measure CBL by calculating the distance between the implant shoulder and the first bone-to-implant contact. All collected data were recorded and analyzed statistically using SPSS software. Descriptive statistics such as mean and standard deviation were calculated. Intergroup comparisons between epicrestal and subcrestal groups were done using the independent t-test, while intragroup comparisons at different time intervals were analyzed using the paired t-test, with a significance level set at p ≤ 0.05. Mean bone height on mesial side of epicrestal and subcrestal bone at baseline was 0.3350±0.7215 and 0.3300±0.07601 respectively. Result was found to be insignificant (p=0.88) when comparing bone height on mesial side of epicrestal and subcrestal bone at baseline. It was clear in table level of bone was better in subcrestal than epicrestal. Mean bone height on distal side of epicrestal and subcrestal bone at baseline was 0.3610±0.11609 and 0.3600±0.10328 respectively. Result was found to be insignificant (p=0.98) when comparing bone height on distal side of epicrestal and subcrestal bone at baseline. It was clear in table level of bone was better in subcrestal than epicrestal (Table 1). Probing pocket depth was zero at baseline in epicrestal and subcrestal bone (Table 2). Mean bone loss on mesial side of epicrestal and subcrestal bone at 6 months was 1.4910±0.19919 and 1.4240±0.10885 respectively. Result was found to be insignificant (p=0.36) when comparing bone loss on mesial side of epicrestal and subcrestal bone at 6 months. It was clear in table that bone loss was maximum in epicrestal (4.5%) in compare to subcrestal. Mean bone loss on distal side of epicrestal and subcrestal bone at 6 months was 1.4170±0.08381 and 1.3180±.27531 respectively. Result was found to be insignificant (p=0.29) when comparing bone loss on distal side of epicrestal and subcrestal bone at 6 months. It was clear in table that bone loss was maximum in epicrestal (6.9%) in compare to subcrestal (Table 3). Mean probing pocket depth on mesial side of epicrestal and subcrestal bone at 6 months was 1.60±0.516 and 1.40±0.516 respectively. Result was found to be insignificant (p=0.39) when comparing probing pocket depth on mesial side of epicrestal and subcrestal bone at 6 months. It was clear in table that probing pocket depth was maximum in epicrestal in compare to subcrestal. Mean probing pocket depth on distal side of epicrestal and subcrestal bone at 6 months was 2.00±0.471 and 1.60±0.516 respectively. Result was found to be insignificant (p=0.09) when comparing probing pocket depth on distal side of epicrestal and subcrestal bone at 6 months. It was clear in table that probing pocket depth was maximum in epicrestal in compare to subcrestal. Mean probing pocket depth on buccal side of epicrestal and subcrestal bone at 6 months was 2.20±0.422 and 2.00±0.667 respectively. Result was found to be insignificant (p=0.43) when comparing probing pocket depth on buccal side of epicrestal and subcrestal bone at 6 months. It was clear in table that probing pocket depth was maximum in epicrestal in compare to subcrestal. Mean probing pocket depth on lingual side of epicrestal and subcrestal bone at 6 months was 1.60±0.516 and 1.40±0.516 respectively. Result was found to be insignificant (p=0.39) when comparing probing pocket depth on lingual side of epicrestal and subcrestal bone at 6 months. It was clear in table that probing pocket depth was maximum in epicrestal in compare to subcrestal (Table 4). Mean bone loss on mesial side of epicrestal and subcrestal bone at 12months was 1.8560±0.19329 and 1.6750±.22858 respectively. Result was found to be insignificant (p=0.07) when comparing bone loss on mesial side of epicrestal and subcrestal bone at 12 months. It was clear in table that bone loss was maximum in epicrestal (9.7%) in compare to subcrestal. Mean bone loss on distal side of epicrestal and subcrestal bone at 12 months was 1.8530±.24432 and 1.7590±.15975 respectively. Result was found to be insignificant (p=0.32) when comparing bone loss on distal side of epicrestal and subcrestal bone at 12 months. It was clear in table that bone loss was maximum in epicrestal (5.1%) in compare to subcrestal (Table 5). Mean probing pocket depth on mesial side of epicrestal and subcrestal bone at 12 months was 1.60±.516 and 1.40±.516 respectively. Result was found to be insignificant (p=0.39) when comparing probing pocket depth on mesial side of epicrestal and subcrestal bone at 12 months. It was clear in table that probing pocket depth was maximum in epicrestal in compare to subcrestal. Mean probing pocket depth on distal side of epicrestal and subcrestal bone at 12 months was 2.00±.667 and 1.80±0.422 respectively. Result was found to be insignificant (p=0.43) when comparing probing pocket depth on distal side of epicrestal and subcrestal bone at 12 months. It was clear in table that probing pocket depth was maximum in epicrestal in compare to subcrestal. Mean probing pocket depth on buccal side of epicrestal and subcrestal bone at 12 months was 1.80±0.422 and 1.60±.516 respectively. Result was found to be insignificant (p=0.35) when comparing probing pocket depth on buccal side of epicrestal and subcrestal bone at 12 months. It was clear in table that probing pocket depth was maximum in epicrestal in compare to subcrestal. Mean probing pocket depth on lingual side of epicrestal and subcrestal bone at 12 months was 1.60±.516 and 1.40±.516 respectively. Result was found to be insignificant (p=0.39) when comparing probing pocket depth on lingual side of epicrestal and subcrestal bone at 12 months. It was clear in table that probing pocket depth was maximum in epicrestal in compare to subcrestal (Table 6). Mean of mesial bone loss in epicrestal at baseline and 6 months was 0.3350 ±0.7215 and 1.4910±.19919 respectively. Results were found to be highly significant (p<0.001) when comparing mean bone loss at 6 months from baseline. It was clear in table that mesial bone loss increase at 6 months in epicrestal. Mean of distal bone loss in epicrestal at baseline and 6months was 0.3610±0.11609 and 1.4170±0.8381 respectively. Results were found to be highly significant (p<0.001) when comparing mean bone loss at 6 months from baseline. It was clear in table that distal bone loss increase at 6 months in epicrestal. Mean of mesial bone loss in subcrestal at baseline and 6months was 0.3300±0.7601 and 1.4240±1.0885 respectively. Results were found to be highly significant (p<0.001) when comparing mean bone loss at 6 months from baseline. It was clear in table that mesial bone loss increase at 6 months in subcrestal. Mean of distal bone loss in subcrestal at baseline and 6 months was 0.3600±0.10328 and 1.3180±0.27531 respectively (Table 7). Results were found to be highly significant (p<0.001) when comparing mean bone loss at 6 months from baseline. It was clear in table that distal bone loss increase at 6 months in subcrestal. Mean of mesial bone loss in epicrestal at baseline and 12 months was 0.3350 ± 0.7215 and 1.8560 ± 1.9329 respectively. Results were found to be highly significant (p<0.001) when comparing mean bone loss at 12 months from baseline. It was clear in table that mesial bone loss increase at 12 months in epicrestal. Mean of distal bone loss in epicrestal at baseline and 12 months was 0.3610±0.11609 and 1.8530±0.24432 respectively. Results were found to be highly significant (p<0.001) when comparing mean bone loss at 12 months from baseline. It was clear in table that distal bone loss increase at 12 months in epicrestal. Mean of mesial bone loss in subcrestal at baseline and 12 months was 0.3300 ± 0.07601 and 1.6750 ± 0.22858 respectively. Results were found to be highly significant (p<0.001) when comparing mean bone loss at 12 months from baseline. It was clear in table that mesial bone loss increase at 12 months in subcrestal. Mean of distal bone loss in subcrestal at baseline and 12 months was 0.3600 ± 0.10328 and 1.7590 ± 0.15975 respectively. Results were found to be highly significant (p<0.001) when comparing mean bone loss at 12 months from baseline. It was clear in table that distal bone loss increase at 12 months in subcrestal (Table 8). Mean of mesial periodontal pocket depth in epicrestal atbaseline and 6 months was 0.0±0.0 and 1.60±0.516 respectively. Results were found to be highly significant (p<0.001) when comparing mean periodontal pocket depth at 6 months from baseline. It was clear in table that mesial periodontal pocket depth increase at 6 months in epicrestal. Mean of distal periodontal pocket depth in epicrestal at baseline and 6 months was .0±.0 and 2.0±0.471 respectively. Results were found to be highly significant (p<0.001) when comparing mean periodontal pocket depth at 6 months from baseline. It was clear in table that distal periodontal pocket depth increase at 6 months in epicrestal. Mean of buccal periodontal pocket depth in epicrestal at baseline and 6 months was 0.0±0.0 and 2.20±0.422 respectively. Results were found to be highly significant (p<0.001) when comparing mean periodontal pocket depth at 6 months from baseline. It was clear in table that buccal periodontal pocket depth increase at 6 months in epicrestal. Mean of lingual periodontal pocket depth in epicrestal at baseline and 6 months was .0±.0 and 1.60±.516 respectively. Results were found to be highly significant (p<0.001) when comparing mean periodontal pocket depth at 6 months from baseline. It was clear in table that lingual periodontal pocket depth increase at 6 months in epicrestal. Mean of mesial periodontal pocket depth in subcrestal at baseline and 6 months was .0±.0 and 1.40±.516 respectively. Results were found to be highly significant (p<0.001) when comparing mean periodontal pocket depth at 6 months from baseline. It was clear in table that mesial periodontal pocket depth increase at 6 months in subcrestal. Mean of distal periodontal pocket depth in subcrestal at baseline and 6 months was .0±.0 and 1.60±.516 respectively. Results were found to be highly significant (p<0.001) when comparing mean periodontal pocket depth at 6 months from baseline. It was clear in table that distal periodontal pocket depth increase at 6 months in subcrestal. Mean of buccal periodontal pocket depth in subcrestal at baseline and 6 months was .0±.0 and 2.00±.667 respectively. Results were found to be highly significant (p<0.001) when comparing mean periodontal pocket depth at 6 months from baseline. It was clear in table that buccal periodontal pocket depth increase at 6 months in subcrestal. Mean of lingual periodontal pocket depth in subcrestal at baseline and 6 months was .0±.0 and 1.40±.516 respectively. Results were found to be highly significant (p<0.001) when comparing mean periodontal pocket depth at 6 months from baseline. It was clear in table that lingual periodontal pocket depth increase at 6 months in subcrestal (Table 10). Mean of mesial bone loss in epicrestal at 6 months and 12 months was 1.4910±.19919 and 1.8560±.19329 respectively. Results were found to be highly significant (p<0.001) when comparing mean bone loss at 12 months from 6 months. It was clear in table that mesial bone loss increase at 12 months in epicrestal. Mean of distal bone loss in epicrestal at 6 months and 12 months was 1.4170±.08381 and 1.8530±.24432 respectively. Results were found to be highly significant (p=0.001) when comparing mean bone loss at 12 months from 6 months. It was clear in table that distal bone loss increase at 12 months in epicrestal. Mean of mesial bone loss in subcrestal at 6 months and 12 months was 1.4240 ±.10885 and 1.6750 ± .22858 respectively. Results were found to be highly significant (p=0.003) when comparing mean bone loss at 12 months from 6 months. It was clear in table that mesial bone loss increase at 12 months in subcrestal. Mean of distal bone loss in subcrestal at 6 months and 12 months was 1.3180±.27531 and 1.7590±.15975 respectively (Table 9). Results were found to be highly significant (p<0.001) when comparing mean bone loss at 12 months from 6 months. It was clear in table that distal bone loss increase at 12 months in subcrestal.

## Results:

Mean of mesial periodontal pocket depth in epicrestal at baseline and 6 months was .0±.0 and 1.60±.516 respectively. Results were found to be highly significant (p<0.001) when comparing mean periodontal pocket depth at 6 months from baseline. It was clear in table that mesial periodontal pocket depth increase at 6 months in epicrestal. Mean of distal periodontal pocket depth in epicrestal at baseline and 6 months was .0±.0 and 2.0±.667 respectively. Results were found to be highly significant (p<0.001) when comparing mean periodontal pocket depth at 6 months from baseline. It was clear in table that distal periodontal pocket depth increase at 6 months in epicrestal. Mean of buccal periodontal pocket depth in epicrestal at baseline and 6 months was .0±.0 and 1.80±.422 respectively. Results were found to be highly significant (p<0.001) when comparing mean periodontal pocket depth at 6 months from baseline. It was clear in table that buccal periodontal pocket depth increase at 6 months in epicrestal. Mean of lingual periodontal pocket depth in epicrestal at baseline and 6 months was .0±.0 and 1.60±.516 respectively. Results were found to be highly significant (p<0.001) when comparing mean periodontal pocket depth at 6 months from baseline. It was clear in table that lingual periodontal pocket depth increase at 6 months in epicrestal. Mean of mesial periodontal pocket depth in subcrestal at baseline and 6 months was .0±.0 and 1.40±.516 respectively. Results were found to be highly significant (p<0.001) when comparing mean periodontal pocket depth at 6 months from baseline. It was clear in table that mesial periodontal pocket depth increase at 6 months in subcrestal. Mean of distal periodontal pocket depth in subcrestal at baseline and 6 months was .0±.0 and 1.80±.422 respectively. Results were found to be highly significant (p<0.001) when comparing mean periodontal pocket depth at 6 months from baseline. It was clear in table that distal periodontal pocket depth increases at 6 months in subcrestal. Mean of buccal periodontal pocket depth in subcrestal at baseline and 6 months was .0±.0 and 1.60±.516 respectively. Results were found to be highly significant (p<0.001) when comparing mean periodontal pocket depth at 6 months from baseline. It was clear in table that buccal periodontal pocket depth increase at 6 months in subcrestal. Mean of lingual periodontal pocket depth in subcrestal at baseline and 6 months was .0±.0 and 1.40±.516 respectively. Results were found to be highly significant (p<0.001) when comparing mean periodontal pocket depth at 6 months from baseline. It was clear in table that lingual periodontal pocket depth increase at 6 months in subcrestal (Table 11). Successful osseointegration of a dental implant alone does not necessarily translate into patient satisfaction. The condition of the peri-implant soft tissues plays a decisive role in the patient's perception of treatment success. Healing around dental implants is influenced by several factors, including systemic health, hard- and soft-tissue architecture, surgical placement and augmentation protocols, prosthetic design and long-term maintenance of the prosthesis. In addition, the position of the implant-abutment junction (IAJ) has been suggested as a determinant of marginal bone alterations. Conventionally, platform-matched implants are positioned with the IAJ either at or above the crestal bone level. Previous investigations have reported that subcrestal implant placement may be associated with greater marginal bone loss when compared to epicrestal placement. Conversely, subcrestal positioning with minimal marginal bone loss has been shown to contribute to stable peri-implant mucosal levels, potentially improving long-term esthetic outcomes. Accordingly, the present clinical study was designed to assess CBL and peri-implant soft-tissue changes following epicrestal and subcrestal implant placement at baseline, 6 months and 12 months after functional loading.

## Discussion:

The present study demonstrated no statistically significant difference in crestal bone height between epicrestal and subcrestal implant groups at baseline, confirming comparable initial conditions. These findings are in strong agreement with Pellicer-Chover et al. (2019) [12], who reported no significant baseline differences between crestal and subcrestal implant placements (p > 0.05), indicating uniformity in pre-operative bone levels. Similarly, Hingsammer *et al.* (2015) [13] observed nearly identical mesial bone levels between epicrestal (0.32 ± 0.08 mm) and subcrestal implants (0.31 ± 0.07 mm), with no statistical significance (p = 0.92). This consistency across studies supports the reliability of proper randomization and comparable alveolar bone morphology prior to implant placement, as also reflected in the current findings. At 6 months, the present study showed slightly greater marginal bone loss in the epicrestal group compared to the subcrestal group, although the difference was not statistically significant. These findings align closely with Linkevicius *et al.* (2020) [14], who reported significantly higher bone loss in epicrestal implants compared to subcrestal implants, attributing this difference to the preservation of biologic width and the advantages of platform switching. Similarly, Froum *et al.* (2018) [15]. demonstrated better bone preservation in subcrestal implants, with complete maintenance of bone-to-implant contact at the crestal level. At 12 months, the present study demonstrated a continued trend of reduced marginal bone loss in subcrestal implants compared to epicrestal implants, although the differences remained statistically non-significant. Additionally, Özer and Acar (2024) [16]. suggested that subcrestal placement reduces biomechanical stress at the crestal bone, which may contribute to slower bone loss progression, as also observed in the present study. Longitudinally, the present study showed significant bone loss from baseline to 6 months and from baseline to 12 months in both groups, followed by a reduction in the rate of bone loss between 6 and 12 months, indicating biological stabilization. These findings are in agreement with Esposito *et al.* (1998) [17], who first described early crestal bone remodeling following implant placement who reported similar patterns of initial bone loss followed by stabilization. Overall, the present findings are largely consistent with existing literature, demonstrating that while both epicrestal and subcrestal implant placements yield comparable clinical outcomes, subcrestal placement tends to show a consistent, though often non-significant and advantage in marginal bone preservation over time.

## Conclusion:

Both epicrestal and subcrestal implant placements showed comparable clinical and radiographic outcomes over 12 months, with no statistically significant intergroup differences. However, subcrestal placement demonstrated a consistent trend of reduced CBL and probing depth, suggesting better peri-implant tissue preservation. Overall, while both techniques are clinically acceptable, subcrestal placement may offer a slight biological advantage, warranting further long-term studies.

## Figures and Tables

**Table 1:** Crestal Bone height at baseline for epicrestal and subcrestal groups (independent t-test)

**BONE LEVEL at baseline**	**GROUP**	**Mean**	**Std. Deviation**	**Mean diff**	**t value**	**p-value**
MESIAL	Epicrestal	0.335	0.07215	0.005	0.151	0.88
	Subcrestal	0.33	0.07601	-1.40%		
DISTAL	Epicrestal	0.361	0.11609	0.001	0.02	0.98
	Subcrestal	0.36	0.10328	-0.20%		
Result-p>0.05insignificant

**Table 2:** Probing pocket depth at baseline for epicrestal and subcrestal

**Probing pocket depth at baseline**	**GROUP**	**Mean**	**Std. Deviation**	**t-value**	**P value**
	Epicrestal	0	0		
MESIAL	Subcrestal	0	0	-	-
	Epicrestal	0	0		
DISTAL	Subcrestal	0	0	-	-
	Epicrestal	0	0		
Buccal	Subcrestal	0	0	-	-
	Epicrestal	0	0		
Lingual	Subcrestal	0	0	-	-

**Table 3:** Comparison of bone loss at 6 months in between epicrestal and subcrestal (independent t test)

**BONE LOSS at 6**	**GROUP**	**Mean**	**Std. Deviation**	**Mean diff (%)**	**t value**	**Pvalue**
months						
MESIAL	Epicrestal	1.491	0.19919	0.6700(4.5%)	0.93	0.36
	Subcrestal	1.424	0.10885			
DISTAL	Epicrestal	1.417	0.08381	0.9900(6.9%)	1.088	0.29
	Subcrestal	1.318	0.27531			
Result-p>0.05insignificant

**Table 4:** Comparison of probing pocket depth at 6 months in between epicrestal and subcrestal (independent t test)

**Probing pocket depth at 6 months**	**GROUP**	**Mean**	**Std. Deviation**	**Meandiff**	**T value**	**p-value**
	Epicrestal	1.6	0.516			
MESIAL	Subcrestal	1.4	0.516	0.2	0.86	0.39
	Epicrestal	2	0.471			
DISTAL	Subcrestal	1.6	0.516	0.4	1.809	0.09
	Epicrestal	2.2	0.422			
Buccal	Subcrestal	2	0.667	0.2	0.81	0.43
	Epicrestal	1.6	0.516			
Lingual	Subcrestal	1.4	0.516	0.2	0.87	0.39
Result-p>0.05insignificant

**Table 5:** Comparison of bone loss at 12 months in between epicrestal and subcrestal (independent t test)

**Bone Loss at 12 months**	**GROUP**	**Mean**	**Std. Deviation**	**Mean diff**	**T value**	**p value**
	Epicrestal	1.856	0.19329			
MESIAL	Subcrestal	1.675	0.22858	0.18100(9.7%)	1.912	0.07
	Epicrestal	1.853	0.24432			
DISTAL	Subcrestal	1.759	0.15975	0.09400(5.1%)	1.018	0.32
Result-p>0.05insignificant

**Table 6:** Comparison of probing pocket depth at 12 months in between epicrestal and subcrestal (independent t test)

**Probing pocket depth at12months**	**GROUP**	**Mean**			**Meandiff**	**Tvalue**	**P-value**
	Epicrestal	1.6	0.516			
MESIAL	Subcrestal	1.4	0.516	0.2	-0.866	0.39
	Epicrestal	2	0.667			
DISTAL	Subcrestal	1.8	0.422	0.2	0.802	0.43
	Epicrestal	1.8	0.422			
Buccal	Subcrestal	1.6	0.516	0.2	0.949	0.35
	Epicrestal	1.6	0.516			
Lingual	Subcrestal	1.4	0.516	0.2	0.866	0.39
Result-p>0.05insignificant

**Table 7:** Comparison of epicrestal and subcrestal bone loss at 6 months from baseline (paired t test)

**GROUP**	**SIDE**	**TIME**	**Mean**	**Std. Deviation**	**Mean diff**	**P-value**
		BASELINE	0.335	0.07215		
	MESIAL	6 MONTHS	1.491	0.19919	1.156	<0.001*
		BASELINE	0.361	0.11609		
Epicrestal	DISTAL	6 MONTHS	1.417	0.08381	1.056	<0.001*
		BASELINE	0.33	0.07601		
	MESIAL	6 MONTHS	1.424	0.10885	1.094	<0.001*
		BASELINE	0.36	0.10328		
Subcrestal	DISTAL	6 MONTHS	1.318	0.27531	0.958	<0.001*
Result-p<0.05significant

**Table 8:** Comparison of epicrestal and subcrestal bone loss at 12 months from baseline (paired t test)

**Group**	**Side**	**Time**	**Mean**	**Std. Deviation**	**Mean diff**	**p-value**
		BASELINE	0.335	0.07215		
	MESIAL	12 MONTHS	1.856	0.19329	1.521	<0.001*
		BASELINE	0.361	0.11609		
EPICRESTAL	DISTAL	12 MONTHS	1.853	0.24432	1.492	<0.001*
		BASELINE	0.33	0.07601		
	MESIAL	12 MONTHS	1.675	0.22858	1.345	<0.001*
SUBCRESTAL		BASELINE	0.36	0.10328		
	DISTAL	12 MONTHS	1.759	0.15975	1.399	<0.001*
Result- p <0.05 significant

**Table 9:** Comparison of epicrestal and subcrestal bone loss at 12 months from 6 months (paired t test)

**Group**	**Side**	**Time**	**Mean**	**Std. Deviation**	**Mean diff**	**p-value**
	MESIAL	6 months	1.491	0.19919	0.365	<0.001*
		12 MONTHS	1.856	0.19329		
		6 months	1.417	0.08381		
EPICRESTAL	DISTAL	12 MONTHS	1.853	0.24432	0.436	0.001*
		6 months	1.424	0.10885		
	MESIAL	12 MONTHS	1.675	0.22858	0.251	0.003*
		6 months	1.318	0.27531		
SUBCRESTAL	DISTAL	12 MONTHS	1.759	0.15975	0.441	<0.001*
Result-p<0.05significant

**Table 10:** Comparison of epicrestal and subcrestal periodontal pocket depth at 6months from baseline (paired t test)

**Group**	**Side**	**Time**	**Mean**	**Std. Deviation**	**Mean diff**	**p-value**
		BASELINE	0	0		
	MESIAL	6 MONTHS	1.6	0.516	1.6	<0.001*
		BASELINE	0	0		
	DISTAL	6 MONTHS	2	0.471	2	<0.001*
		BASELINE	0	0		
EPICRESTAL	BUCCAL	6 MONTHS	2.2	0.422	2.2	<0.001*
		BASELINE	0	0		
	LINGUAL	6 MONTHS			1.6	<0.001*
SUBCRESTAL	MESIAL	BASELINE	0	0	1.4	<0.001*
		6 MONTHS	1.4	0.516		
		BASELINE	0	0		
	DISTAL	6 MONTHS	1.6	0.516	1.6	<0.001*
		BASELINE	0	0		
	BUCCAL	6 MONTHS	2	0.667	2	<0.001*
		BASELINE	0	0		
	LINGUAL	6 MONTHS	1.4	0.516	1.4	<0.001*
Result-p<0.05significant

**Table 11:** Comparison of epicrestal and subcrestal periodontal pocket depth at 12 months from baseline (paired t test)

**Group**	**Side**	**Time**	**Mean**	**Std. Deviation**	**Mean diff**	**p value**
	MESIAL	6 MONTHS	1.6	0.516	1.6	<0.001*
		BASELINE	0	0		
EPICRESTAL	DISTAL	6 MONTHS	2	0.667	2	<0.001*
		BASELINE	0	0		
	BUCCAL	6 MONTHS	1.8	0.422	1.8	<0.001*
		BASELINE	0	0		
	LINGUAL	6 MONTHS	1.6	0.516	1.6	<0.001*
		BASELINE	0	0		
	MESIAL	6 MONTHS	1.4	0.516	1.4	<0.001*
		BASELINE	0	0		
	DISTAL	6 MONTHS	1.8	0.422	1.8	<0.001*
SUBCRESTAL		BASELINE	0	0		
	BUCCAL	6	1.6	0.516	1.6	<0.001*
		BASELINE	0	0		
	LINGUAL	6 MONTHS	1.4	0.516	1.4	<0.001*
Result-p<0.05significant
